# phyloSkeleton: taxon selection, data retrieval and marker identification for phylogenomics

**DOI:** 10.1093/bioinformatics/btw824

**Published:** 2017-01-05

**Authors:** Lionel Guy

**Affiliations:** Department of Medical Biochemistry and Microbiology, Uppsala University, Uppsala, Sweden

## Abstract

**Summary:**

With the wealth of available genome sequences, a difficult and tedious part of inferring phylogenomic trees is now to select genomes with an appropriate taxon density in the different parts of the tree. The package described here offers tools to easily select the most representative organisms, following a set of simple rules based on taxonomy and assembly quality, to retrieve the genomes from public databases (NCBI, JGI), to annotate them if necessary, to identify given markers in these, and to prepare files for multiple sequence alignment.

**Availability and Implementation:**

phyloSkeleton is a Perl module and is freely available under GPLv3 at https://bitbucket.org/lionelguy/phyloskeleton/.

## 1 Introduction

Many studies (e.g. Pick *et al.*, 2010) have highlighted the importance of the effect of taxon sampling on recovering the correct species tree with phylogenomics methods, although the relative benefits of adding more taxa or more genes has been hotly debated (e.g. Philippe *et al.*, 2011; Rosenberg and Kumar, 2003; Zwickl and Hillis, 2002). Thanks to the wealth of publicly available sequence data, the affordability of DNA sequencing, and the rise of single-cell and metagenomics methods, the bottleneck in establishing phylogenies is now computational. To keep phylogenetic inference tractable, researchers face a trade-off between number of taxa and number of marker genes to include.

One of the frequent aims of phylogenomic studies is to place novel, unknown organisms in their phylogenetic context, using a backbone tree composed of well-known organisms. To achieve this, or to resolve a particular region of the tree of life, a common solution is to obtain a denser sampling close to the nodes of interest, and less dense further away. In practice, this is often achieved by a recursive process:

1. Select representative genomes to include.
2. Retrieve the corresponding genomes and, if available, proteomes or else annotate the genomes.
3. Identify orthologs of selected marker genes in each proteome.
4. Align the sequences for each marker separately.
5. Concatenate the alignment, tracking protein names and ids.
6. Infer a phylogeny. Upon tree inspection, if the density of taxa at the place of interest is not good enough, go back to (1).

The whole process is often repeated many times: first, trees are computed with faster phylogenetic methods, e.g. FastTree (Price *et al.*, 2010), until the right sampling density is achieved at the right place, when more sensitive algorithms like RAxML (Stamatakis, 2014) or PhyloBayes (Rodrigue and Lartillot, 2014) can be used.

Identifying orthologs (step 3) is a difficult algorithmic problem, and accurately excluding paralogous sequences generally requires visual inspection of each single-gene tree. Multiple sequence alignment (step 4) and phylogenetic inference (step 6) are the most computationally intensive and are often the bottleneck of the analysis.

However, the other steps are often tedious, requiring long hands-on time searching databases and keeping track of protein and organisms names, slowing down the whole process. Step 1, selecting representative genomes, can be especially problematic when sampling well-studied taxa (e.g. the Enterobacteriaceae) counting thousands of available genomes.

Many software packages aim at automating different parts of the phylogenomics process, but to the best of my knowledge, none covers it all or addresses the automated selection of representative taxa.

For example, Agalma (Dunn *et al.*, 2013) automates the annotation of transcriptome data, the alignment of homologous marker sets and performs a preliminary phylogeny, but relies on data provided by the user.

BIR (Kumar *et al.*, 2015) and PhlyoTreePruner (Kocot *et al.*, 2013) attempt to automatically identify orthologs, but they require the user to provide curated sets of markers or single-gene trees, respectively.

Phyla-AMPHORA (Wang and Wu, 2013) gathers phylum-level markers, but does not provide means to vary the density of taxon sampling. It would be interesting to include phylum-level alignments in the phyloSkeleton pipeline, but unfortunately, Phyla-AMPHORA has not been updated since its publication.

PhyloSift (Darling *et al.*, 2014) and CheckM (Parks *et al.*, 2015), intended for metagenomics datasets, have the possibility to place sequences into a fixed reference backbone tree.

MicrobeDB (Langille *et al.*, 2012) allows the user to maintain a local database of publicly available and own genome sequences, easing the burden of maintaining a backbone tree.

The purpose of phyloSkeleton is to automate steps 1 and 2, picking up the best representative at a variable density as decided by the user, retrieving the genomic data, annotating genes if necessary; it also facilitates step 3, identifying orthologs, by automatically preparing single-gene trees. Finally, phyloSkeleton automates the concatenation of single-gene alignments, and provides useful tools to visualize trees.

So far, phyloSkeleton is aimed primarily at prokaryotic genomes, but could potentially be used for eukaryotic ones.

## 2 Methods

### 2.1 Selection of representative genomes

The user first retrieves lists of available genomes from NCBI (Genbank) and, optionally, from the Joint Genomic Institute (JGI; IMG database). The selection of representative genomes is based on a set of simple taxonomic rules: at a specific higher level (e.g. class), select one representative per lower level (e.g. genus). These rules can be combined to achieve the right sampling density at the right place, for example sampling at species level in the order of interest, and at class level in the selected outgroup phylum. The user also has the possibility to add their own data.

The selection algorithm selects the best representative, first preferring (i) a reference or (ii) a representative genome if they are available in NCBI’s Entrez genome collection. Then, the assembly level and the source are considered, looking in decreasing order of preference at (i) complete or (ii) chromosome level assemblies at NCBI, (iii) finished projects at JGI, (iv) chromosome with gaps, (v) scaffolds or (vi) contigs assembly level at NCBI and at (vi) permanent draft or (vii) draft at JGI. Lastly, the largest genomes are favored.

The genomes and, eventually, the proteomes of the selected representatives are automatically retrieved from NCBI and/or JGI. Genomes for which no proteome is available are annotated with prodigal (Hyatt *et al.*, 2010) or prokka (Seemann, 2014).

### 2.2 Marker selection

All genomes are screened for marker genes that will be used for the concatenated phylogeny. The user provides a set of HMM profiles corresponding to these markers. Three generic sets, one consisting of 15 ribosomal protein genes, one bacteria- and one archaea-specific (Rinke *et al.*, 2013), are shipped with the software. HMMER (Eddy, 2011) is used to identify the best matches. If there is more than one significant match per proteome, a warning is raised and a single-gene phylogeny for this specific marker is prepared, to help the user selecting the correct paralog.

### 2.3 Other tools

After marker identification, phyloSkeleton gathers the protein sequences in fasta files, and, upon alignment, concatenate the alignments. It also contains scripts to facilitate analyzing large trees by adding colors and group names, in conjunction with FigTree (Andrew Rambaut, http://tree.bio.ed.ac.uk/software/figtree/).

## 3 Conclusion

PhyloSkeleton gathers genome sequences to infer a phylogenetic tree with variable taxon sampling density, following simple rules based on taxonomy and genome assembly quality. It is especially useful to place a novel, unknown organism in a backbone tree, or to resolve a particular region of a large tree, or to explore the monophyly of certain taxa.

It allows the user to quickly perform many iterations of the phylogenomic process: changing the selection rules to modify taxon sampling density is the only manual step once the initial run has completed.

The software, released under GPLv3, comes with a comprehensive manual, a complete tutorial and a test data set, available at https://bitbucket.org/lionelguy/phyloskeleton.
